# Notes and News

**Published:** 1896-10-03

**Authors:** 


### NOTES AND NEWS.

(Collected and Communicated.)

The Harveian Oration will be delivered by Dr. J. Frank Payne on Monday, October 19th, at four p.m.

Dr. Sydney Martin has been appointed Croonian Lecturer at the College of Physicians for the ensuing year.

The Salford Royal Hospital has received the sum of £6,250, being an instalment of a legacy under the will of the late Mr. Samuel Weston.

The Empress Frederick celebrated the anniversary of her betrothal to the late Emperor Frederick on September 29th by laying the foundation-stone of the Kronberg District Hospital.

Dr. Garrett Horder convened a meeting at the office of the Medical Defence Union on Thursday, October 1st, to promote a Hospital Reform Association with the view of abating hospital abuse.

The foundation stone of the Passmore Edwards' Convalescent Home at Cranbrook, for the use of the patients of the Metropolitan Hospital, will be laid on Wednesday, October 14th, at a quarter past three, by Mrs. Cornwallis.

The Harrogate Cottage Hospital has just been reopened, after having been enlarged and extended. The new wing, named the "Clarence" wing, gives accommodation for seven children. The alterations and additions have cost £2,800.

The new Rustington Convalescent Home is now completed, and will shortly be opened. It has been erected by Mr. Henry Harben at a cost of £30,000, and is intended for the use of the working classes in North-West London. The home faces the sea, and will accommodate forty patients.

Bermondsey is leading the way with a new sanitary precaution. Dr. Dixon, the medical officer of health, has been instructed to inform the librarian where infectious diseases exist, in order that books may not be issued to the inmates of such houses, and thus become sources of contagion.

Dr. Samuel Wilks, President of the Royal College of Physicians, has accepted the office of consulting physician to the Great Northern Central Hospital, Holloway Road.

We are informed that in consequence of the death of Sir John Eric Erichsen, the dinner of the Old and Present Medical Students of University College will not be held this year.

A cottage hospital has just been opened at Oban. It was founded by Mrs. Parr, of Killichronan, who opened the completed building recently, and who gave £2,600 to defray the cost of erection. The hospital contains two wards with five beds in each and two private wards.

There is to be a new hospital for Newport and Monmouth. The committee have offered two prizes of £100 and £50 for designs, the first prize merging into the premium of the successful architect. The expenditure is to be limited to £16,000, including furniture and fittings.

Dr. Yersin, whose reputed discovery of a serum which cures the plague we mentioned some time past, has continued his practice, and is quite satisfied with the results. He holds that when the plague is treated by his method immediately, a cure is effected in twelve hours.

The crematorium which has been established at Liverpool has the outward appearance of a Gothic church, with a tower standing a little to one side. Beyond the chapel is an anti-chamber, and in the wall of this is the aperture leading to the furnace. The furnace is entirely screened from view.

Mr. Ben Evans' offer to provide the Swansea Hospital with a new operating theatre has been accepted, and a site chosen on the north side of the hospital. Several members of the committee have been deputed to visit other institutions to inspect existing operating theatres.

Miss Mary Cannington, of Cotham, who intended to leave £10,000 to the Bristol Royal Infirmary, has recently presented the institution with an equivalent sum invested in 2¾ per cent. Consols. She has made certain stipulations, one of which is that part of the money shall be devoted to the maintenance of a ward to be called after her uncle, James Palmer.

A reception will be held by the Society for the Study of Inebriety on Thursday, October 8th, at a quarter to four p.m., in the rooms of the Medical Society of London, 11, Chandos Street. The reception will be held in honour of Professor G. von Bunge, of the University of Basle. A paper on "The Opium Habit: Some Points in Diagnosis and Prognosis," will be read by Dr. W. Huntly, late of Rajpootana.

The New Convalescent Home at Grange-over-Sands, built for the use of members of the friendly societies of the north-eastern counties, is now nearly ready for occupation. It stands upon a well-wooded slope 100 ft. above sea level, and commands delightful views. The Home is designed on the pavilion plan. At present only the administration block and one wing will be built. The dining hall will seat seventy persons. The day room, which faces the sea, leads on to a verandah. A smoking-room adjoins the day-room. The cost of the Home is estimated at £3,000.

